# Future leader to watch – Nathan Harmston

**DOI:** 10.1242/bio.059321

**Published:** 2022-04-04

## Abstract

First Person is a series of interviews with the authors of a selection of papers published in Biology Open, helping early-career researchers promote themselves alongside their papers. Nathan Harmston is last author on ‘ The importance of considering regulatory domains in genome-wide analyses ­– the nearest gene is often wrong!’, published in BiO. Nathan is Assistant Professor of Computational Biology at Yale NUS College, Singapore, investigating the rules and features of how gene expression is regulated in both development and disease.

## What is your scientific background and the story of how you got to where you are today?

I studied computer science at the University of Nottingham and in my final year took a course on computational biology. This course made me realize that computational biology was something that I would be really interested in pursuing. My first exposure to computational biology research was during a summer internship at MyCIB at the University of Nottingham, where I was building tools for high-performance computing and starting with some initial analysis of DNA sequences. I continued with a masters in bioinformatics and systems biology at Imperial College London. I remained at Imperial College to pursue a PhD, under the supervision of Professors Michael Stumpf and Wendy Filsell, to develop computational methods for extracting networks and models from text. I then moved to work with Professor Boris Lenhard at the MRC CSC in London and spent my first post-doc investigating the evolution of regulatory domains and conserved non-coding elements in genomes. From there, I moved to Duke-NUS in Singapore to work with Professor Enrico Petretto on understanding gene regulation and expression in human disease. In 2019, I started as an assistant professor at Yale-NUS, where I continued my research program studying gene regulation and genome evolution.

**Figure:**
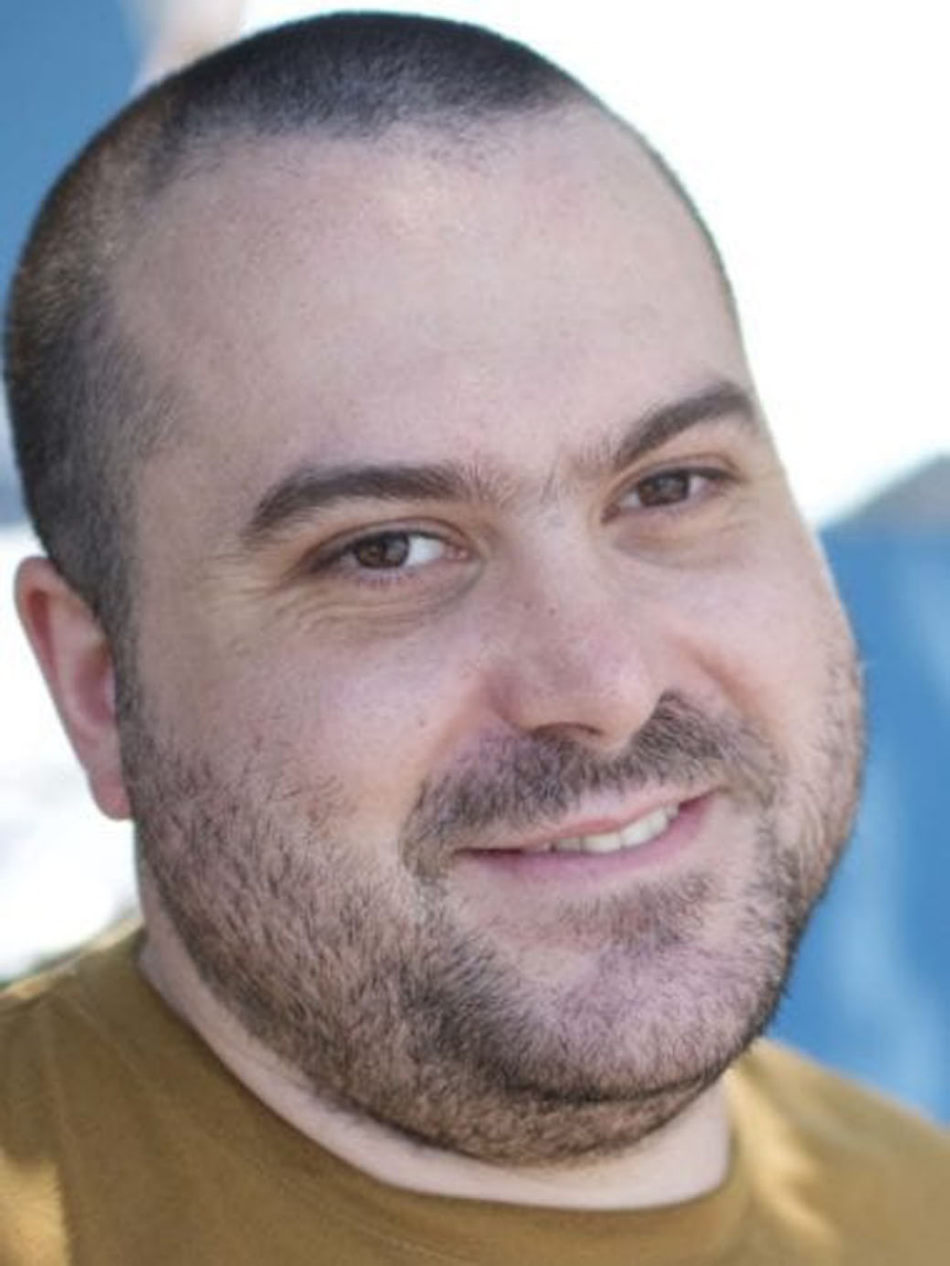
Nathan Harmston

## What is the most important take-home message of your review?

Often when researchers study the genome, they're trying to identify regions that are involved in turning a specific gene on or off, or that are associated with a specific trait. But once these regions have been found, the question is always ‘what gene does this actually effect?’. My review focuses on how researchers link variants to genes, and discusses the many studies, from multiple angles, which highlight that in order to do this well, we really need to consider the 3D structure of folded DNA and not just focus on the nearest gene.

## What has surprised you the most while researching this review?

The ways in which different types of studies (single locus, genome-wide, comparative genomics) and different technologies can all come together to really show quantitatively that nearest gene assignment is often wrong.

## What do you feel is the most important question that needs to be answered to move the field forward?

There are so many outstanding questions in gene regulation out there, especially when thinking about regulatory grammars and long-range regulation. But I think it's really important to understand how specificity is encoded in the DNA sequence of a regulatory element. Why is it that this enhancer specifically regulates that gene? Why is it that this regulatory element is only active in that specific context? Why is this gene a target of a signaling pathway in this context but not another?

## What changes do you think could improve the professional lives of early-career researchers?

I feel that there is really a need for good consistent mentorship coupled with clear expectations and support from the university/institute.

## What's next for you?

To continue asking (and hopefully answering) interesting questions about gene regulation, and to help students develop and succeed as researchers.

## References

[BIO059321C1] Chua, E. H. Z., Yasar, S. and Harmston, N. (2022). The importance of considering regulatory domains in genome-wide analyses ­– the nearest gene is often wrong!. *Biology Open* 11 bio.059091. 10.1242/bio.059091 PMC9002814 35377406

